# Design, synthesis, and evaluation of novel arecoline-linked amino acid derivatives for insecticidal and antifungal activities

**DOI:** 10.1038/s41598-024-60053-2

**Published:** 2024-04-24

**Authors:** Chaohai Pang, Yuan Xu, Xionghui Ma, Shuhuai Li, Shengfu Zhou, Hai Tian, Mingyue Wang, Bingjun Han

**Affiliations:** 1 Hainan Provincial Key Laboratory of Quality and Safety for Tropical Fruits and Vegetables, Key Laboratory of Quality and Safety Control of Subtropical Fruits and Vegetables, Analysis and Test Center, Chinese Academy of Tropical Agricultural Sciences, Ministry of Agriculture and Rural Affairs, Haikou, 571101 China; 2 Engineering Research Center of Tropical Medicine Innovation and Transformation of Ministry of Education, International Joint Research Center of Human-machine Intelligent Collaborative for Tumor Precision Diagnosis and Treatment of Hainan Province, Hainan provincial key laboratory of research and development on tropical herbs, School of Pharmacy, Hainan Medical University, Haikou, 571199 China; 3 Shenzhen Bay Laboratory, BayRay Innovation Center, Shenzhen, 518000 China

**Keywords:** Arecoline, Derivatives, Insecticidal activity, Fungicidal activity, Molecular docking, Chemical modification, Biochemistry, Medical research, Chemistry

## Abstract

A series of arecoline derivatives with amino acid moieties were designed and synthesised using an acylamide condensation strategy, taking arecoline as the foundational structure. The insecticidal efficacy of these compounds against *Aphis craccivora* and *Tetranychus cinnabarinus* was evaluated. Notably, derivatives **3h** and **3i** demonstrated superior insecticidal activity compared with arecoline. Additionally, **3h** and **3i** showed good fungicidal effectiveness against two types of plant fungi. Moreover, molecular docking analyses suggested that **3h** and **3i** could affect the nervous systems of *A. craccivora* and *T. cinnabarinus* by binding to neuronal nicotinic acetylcholine receptors. These findings suggest that compounds **3h** and **3i** represent promising leads for further development in insecticide and fungicide research.

## Introduction

The long-term and indiscriminate use of synthetic agrochemicals has led to increasingly severe issues with pesticide residues and resistance. Pesticides persist and accumulate in the environment and can enter the human body through various pathways, posing potential threats to human health and life. Moreover, the problem of pesticide resistance intensifies the emergence of super pests, directly disrupting ecosystem balance^1–3^. Despite the critical role of pesticides in securing agricultural production and product quality, the challenges posed by residues, pest resistance, and regrowth have become significant concerns for the global agricultural sector^4–6^. Addressing these challenges calls for the exploration and development of novel insecticides featuring new targets and modes of action. Natural products, known for their broad-spectrum insecticidal and fungicidal activities, serve as valuable resources for creating innovative pesticides in agriculture^7–10^. Alkaloids, a notable class of natural insecticides, exemplified by matrine^11^ and nicotine^12^, have found success in the development and application of novel botanical pesticides.

Arecoline, an alkaloid derived from the areca nut, exhibits diverse biological activities, including antimicrobial^13^, anti-inflammatory^14^, antiparasitic, and anthelmintic effects^15^. Research^16^ has shown that arecoline has certain insecticidal activity against *Plutella xylostell*. Additionally, arecoline has a certain acetylcholinesterase (AChE) inhibitory activity^16^, which is similar to the main insecticidal mechanism of organophosphates and carbamates pesticides. Also, some research studies^17,18^ have shown that arecoline is a muscarinic acetylcholine receptor agonist, with cholinergic effects. However, research on arecoline has predominantly focused on medical applications^19–21^, with limited exploration in agriculture. Thus, a thorough investigation into arecoline’s insecticidal potential is crucial for fully leveraging arecoline plant resources and discovering new lead compounds for botanical insecticides.

Amino acids, known for their special permeability, low toxicity, enzyme catalytic specificity, participate in multiple chemical reactions^22^, and are promising for developing lead compounds with ideal biological activity when introduced into natural product precursors^23,24^. Notably, various agricultural chemicals, including insecticides, fungicides, and herbicides, incorporate amino acids and their derivatives, such as fluoroacetamide, chlorobenzamide, glyphosate^25^, and bromoxynil^26^. Structure–activity relationship studies have highlighted the critical role of unique amino acid structural fragments in exerting insecticidal activities. For instance, the interaction between amino acid residues and the AChR target of pests can significantly enhance insecticidal activity^27^. Recent modifications of monomers with amino acids and their derivatives as functional fragments have led to pesticides with enhanced insecticidal activity^25,28–30^. For example, Zhou et al.^28^ designed 18 novel *N*-phenylacetamide derivatives by incorporating alanine and serine, demonstrating significant insecticidal activity against *Plutella xylostella*. Mao et al.^29^ designed and synthesised novel *N*-pyridylpyrazolecarboxamide derivatives containing amino acid esters, based on the commercial insecticide, chlorfenapyr. The target derivatives with the methacrylic acid substructure exhibited strong insecticidal activity against diamondback and oriental fruit moths. Zhu et al.^30^ synthesised 69 amino acid-substituted 4-ethynylphenylsulfonyl amide derivatives and evaluated their insecticidal activities against third-instar *S. litura* and *Chilo suppressalis*. Derivatives D16, D18, and D19 showed more than 14-fold higher insecticidal activity than the commercial insecticide avermectin V. These findings preliminarily confirm the potential of amino acids and their derivatives to enhance the insecticidal activity of monomeric compounds.

Building on this theoretical and research foundation, the current study introduced nine amino acids into the molecular structure of arecoline using acylation condensation reaction, with hexafluorophosphate azabenzotriazole tetramethyl uronium (HATU) as the condensing agent, to design and synthesise a series of novel arecoline derivatives with amino acid bonds. This synthetic strategy allows for the production of high-purity target derivatives through simple solvent extraction and recrystallisation, effectively bypassing the laborious and time-consuming processes typically associated with conventional and column chromatography. The insecticidal and fungicidal activities of these target derivatives were evaluated, and molecular docking was employed to explore the preliminary mechanisms of action of compounds **3h** and **3i**. This work not only diversifies and expands the chemical structure of arecoline but also offers new insights and viable candidates for developing novel arecoline-based insecticides and fungicides.

## Results and discussion

### Chemistry

**Figure 1:**
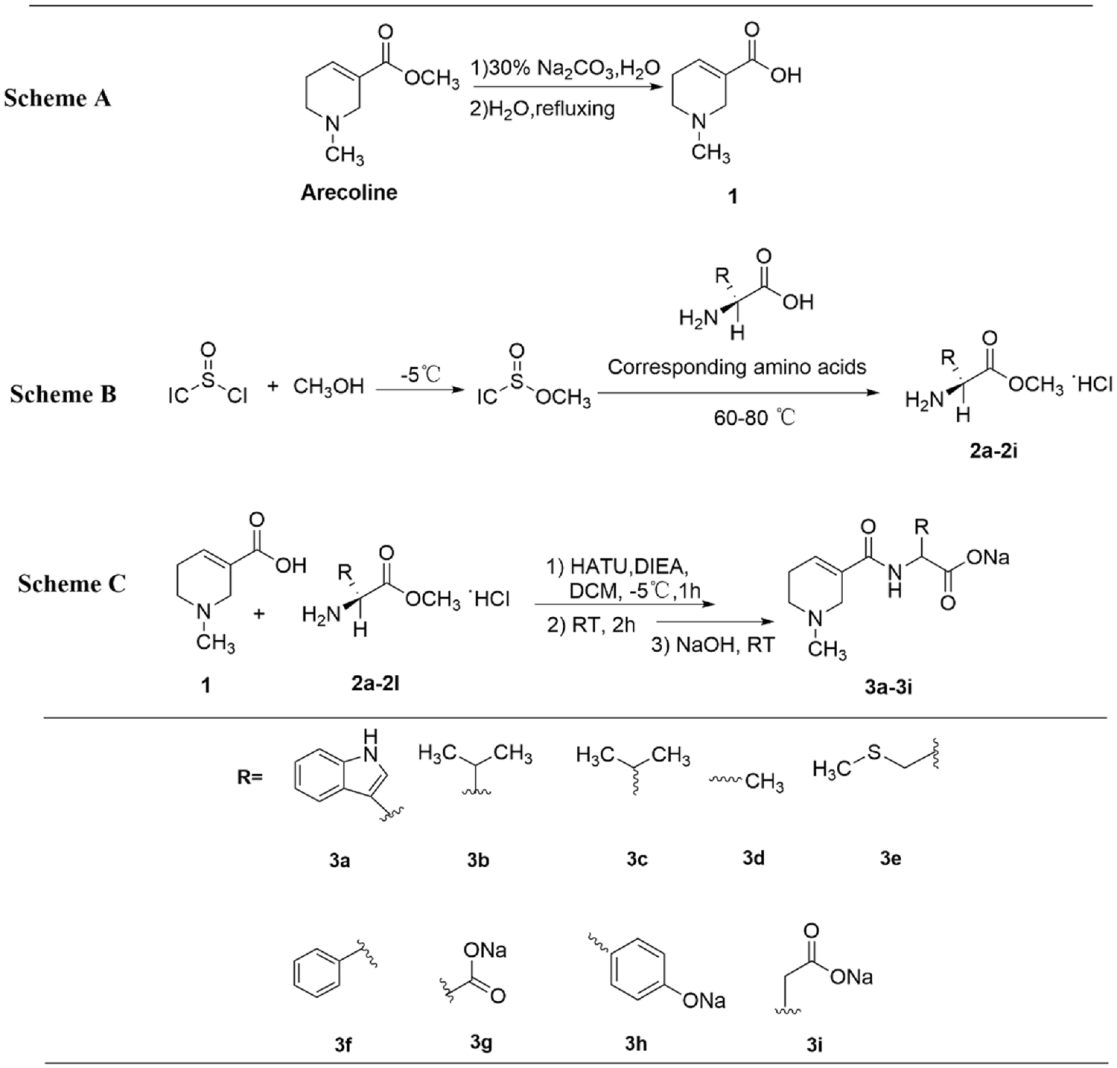
Synthesis procedures of compounds** 3a–3i**.

The synthesis pathway for the target compounds is depicted in Fig. 1. Scheme A illustrates that compound **1** was derived from arecoline through a hydrolysis reaction. The essential intermediates **2a–2l** were synthesised following a method previously described in the literature^31^, utilising sulfifinyl chloride as detailed in Scheme B. These intermediates, **2a–2l,** underwent purification via recrystallisation in methanol and anhydrous ether, effectively preserving the amino acid carboxyl terminal with a methyl ester.

In conditions maintained under an ice bath, employing HATU as the condensing agent, DIEA as the base, and DCM as the solvent, compounds **1** and **2** underwent amide coupling to yield their respective crude products. Subsequently, these crude products were hydrolysed with NaOH at room temperature to generate the corresponding sodium salts, and the final target compounds **3a–3l** of enhanced purity were acquired through recrystallisation in a water–acetone mixture (Scheme C). Notably, this synthesis process allows for the attainment of target compounds with superior purity by adeptly conducting solvent extraction, hydrolysis, and recrystallisation, tailored to the specific chemical properties of the substrates and products. As shown in Fig. S1, the ^1^H-NMR spectrum results of 3h showed that some impurities can be significantly removed after recrystallization treatment to achieve the purpose of improving purity. This strategy effectively circumvents the labour intensive and time-consuming purification methods, such as column chromatography.

The ^1^H-NMR, ^13^C-NMR, and high-resolution mass spectra for the target compounds **3a–3i** are meticulously detailed in the Supporting Information.

### Insecticidal biological activity

The insecticidal efficacy of the target compounds against *A. craccivora* and *T. cinnabarinus* is presented in Tables 1, 2 and 3. Imidacloprid and avermectin served as the reference standards. As indicated in Table 1, only compounds **3h** and **3i** achieved more that 50% mortality against *A. craccivora* and *T. cinnabarinus* at a concentration of 400 mg/L, with efficacy diminishing at reduced concentrations (200 mg/L). Furthermore, compounds **3h** and **3i** exhibited mortality rates surpassing that of the precursor compound, arecoline, at 400 mg/L. Compounds **3h** (R = sodium phenolate) and **3i** (R = sodium propionate), characterised by their strong electronegativity, demonstrated notable insecticidal potency. Hence, we tentatively propose that the inclusion of highly electronegative groups is beneficial for enhancing insecticidal activity. Furthermore, the LC_50_ values of compounds **3h** and **3i** against *A. craccivora* and *T. cinnabarinus* were determined (Tables 2, 3), revealing LC_50_ values of 394.66 mg/L and 208.01 mg/L for **3h** and 275.28 mg/L and 257.46 mg/L for **3i**, respectively. Overall, The LC_50_ values of **3h** and **3i** were lower than those for the parent compound, arecoline (634.19/315.35 mg/L), yet higher than those for imidacloprid and avermectin (7.75/3.88 mg/L).

**Table 1:** Mortality rates of compounds **3a–3i** and arecoline against *A. craccivora* and *T. cinnabarinus*.

Compd	The 48 h mortality (%) at a concentration of (mg/L)					
	*A. craccivora*			*T. cinnabarinus*		
	800	400	200	800	400	200
**3a**	40.0 ± 3.3	30.0 ± 0.0	26.7 ± 5.7	28.9 ± 1.9	18.9 ± 1.9	11.1 ± 5.0
**3b**	53.3 ± 6.7	36.7 ± 3.3	23.3 ± 0.0	38.9 ± 9.6	30.0 ± 3.3	22.2 ± 6.9
**3c**	50.0 ± 13.3	33.3 ± 8.8	20.0 ± 0.0	43.3 ± 5.7	28.9 ± 1.9	17.8 ± 5.0
**3d**	60.0 ± 3.3	40.0 ± 8.8	20.0 ± 0.0	48.9 ± 1.9	34.4 ± 3.8	21.1 ± 5.0
**3e**	30.0 ± 8.8	20.0 ± 0.0	13.3 ± 0.0	37.8 ± 3.8	27.8 ± 1.9	15.6 ± 5.0
**3f**	43.3 ± 5.8	26.7 ± 5.8	16.7 ± 3.3	51.1 ± 1.9	35.6 ± 1.9	25.6 ± 1.9
**3g**	63.3 ± 12.0	33.3 ± 6.7	30.0 ± 3.3	54.4 ± 7.7	42.2 ± 3.8	30.0 ± 3.3
**3h**	86.7 ± 5.8	56.7 ± 5.8	26.7 ± 5.8	82.2 ± 5.0	63.3 ± 3.3	50.0 ± 6.7
**3i**	93.3 ± 5.8	63.3 ± 5.8	36.7 ± 5.8	70.0 ± 3.3	57.8 ± 6.9	44.4 ± 5.1
Arecoline	60.0 ± 3.3	33.3 ± 0.0	30 ± 5.8	61.1 ± 1.9	54.4 ± 1.9	43.3 ± 5.8

Values are the means ± SDs (n = 3).

**Table 2:** LC_50_ Values of Compounds **3a–3i** and Imidacloprid against *A. craccivora*.

Compd	LC_50_ (mg/L)	Regression equation	R
**3a**	> 800	Y = 1.9807 + 1.0030x	0.9651
**3b**	> 800	Y = 0.2434 + 1.5253x	0.9981
**3c**	> 800	Y = 0.7785 + 1.3184x	0.9966
**3d**	598.16	Y = 0.5578 + 1.5997x	0.9722
**3e**	> 800	Y = 0.2154 + 1.4415x	0.9663
**3f**	> 800	Y = 0.0935 + 1.5485x	0.9729
**3g**	545.21	Y = 1.5152 + 1.2734x	0.9687
**3h**	394.66	Y = -0.5038 + 1.7224x	0.9333
**3i**	275.28	Y = 0.6462 + 1.7845x	0.9124
Arecoline	634.19	Y = 1.2393 + 1.3420x	0.9026
Imidacloprid^a^	7.75	Y = 3.6369 + 1.5327x	0.9793

Analysis by GraphPad Prism 8.3.0.538.

^a^Commercial agricultural insecticide used for comparison.

**Table 3:** LC_50_ Values of Compounds **3a–3i** and Avermectin against *T.cinnabarinus*.

Compd	LC_50_ (mg/L)	Regression equation	R
**3a**	> 800	Y = 0.5403 + 1.3637X	0.9763
**3b**	> 800	Y = 0.4493 + 1.3951X	0.9749
**3c**	> 800	Y = − 0.3845 + 1.6629X	0.9766
**3d**	> 800	Y = 0.4694 + 1.5922X	0.9944
**3e**	> 800	Y = 0.4519 + 1.4984X	0.9768
**3f**	624.47	Y = 0.6590 + 1.5528X	0.9880
**3g**	510.73	Y = 0.8771 + 1.5224X	0.9834
**3h**	208.01	Y = 1.6548 + 1.4431X	0.9923
**3i**	257.46	Y = 1.4698 + 1.4644X	0.9673
Arecoline	315.35	Y = 1.8602 + 1.2565X	0.9673
Avermectin^a^	3.88	Y = 3.8337 + 1.9804X	0.9894

Analysis by GraphPad Prism 8.3.0.538.

^a^Commercial agricultural insecticide used for comparison.

### Fungicidal activity

The fungicidal activities of the target compounds **3a–3i,** alongside standard controls (arecoline and chlorothalonil) against five types of plant fungi were evaluated and are summarised in Table 4. Among the compounds tested, **3e** demonstrated inhibitory rates of 39% against *Colletotrichum gloeosporioides* and 49% against *Botrytis cinerea*, and its activity level is equivalent to that of arecoline (41% and 48%, respectively). Compounds **3h** and **3i** exhibited enhanced inhibitory activity against *C. gloeosporioides* (54%/48%) and *B. cinerea* (70%/64%) compared with arecoline (41%/48%). On the whole, the antifungal activities of compounds **3e**, **3h** and **3i** against against *C. gloeosporioides* and *B. cinerea* were outstanding, warranting further investigation as potential fungicide candidates.

**Table 4:** In vitro fungicidal activities of** 3a–3i,** arecoline and chlorothalonil against five kinds of fungi.

Compd	Fungicidal activities (%) at 50 mg/L				
	P.O.C	C.G.162	F. G. S	C.F.63	B.C
**3a**	11 ± 2	9 ± 2	8 ± 1	9 ± 1	9 ± 2
**3b**	8 ± 1	10 ± 1	8 ± 1	10 ± 2	8 ± 1
**3c**	10 ± 1	11 ± 3	7 ± 2	9 ± 1	10 ± 2
**3d**	11 ± 3	10 ± 1	10 ± 2	12 ± 2	11 ± 1
**3e**	11 ± 1	39 ± 2	14 ± 2	12 ± 1	49 ± 3
**3f**	10 ± 2	18 ± 2	16 ± 2	12 ± 1	19 ± 2
**3g**	12 ± 2	23 ± 1	21 ± 1	15 ± 3	21 ± 1
**3h**	12 ± 1	54 ± 2	14 ± 3	16 ± 1	70 ± 1
**3i**	18 ± 1	48 ± 1	15 ± 1	23 ± 1	64 ± 2
Arecoline	18 ± 2	41 ± 2	12 ± 2	18 ± 1	48 ± 1
Chlorothalonil^a^	80 ± 2	59 ± 1	61 ± 1	60 ± 2	95 ± 2

Values are means ± SDs (n = 3).

*P.O.C Pyricularia oryae* Cav, *C.G.162 Colletotrichum gloeosporioides*, *F.G.S* FusaHum graminearum Sehw, *C.F.163 Colletotrichum fragariae*, *B.C Botrytis cinerea*, prominent activity data are presented in blue color.

^a^Commercial agricultural fungicide used for comparison.

### Docking simulation

**Figure 2:**
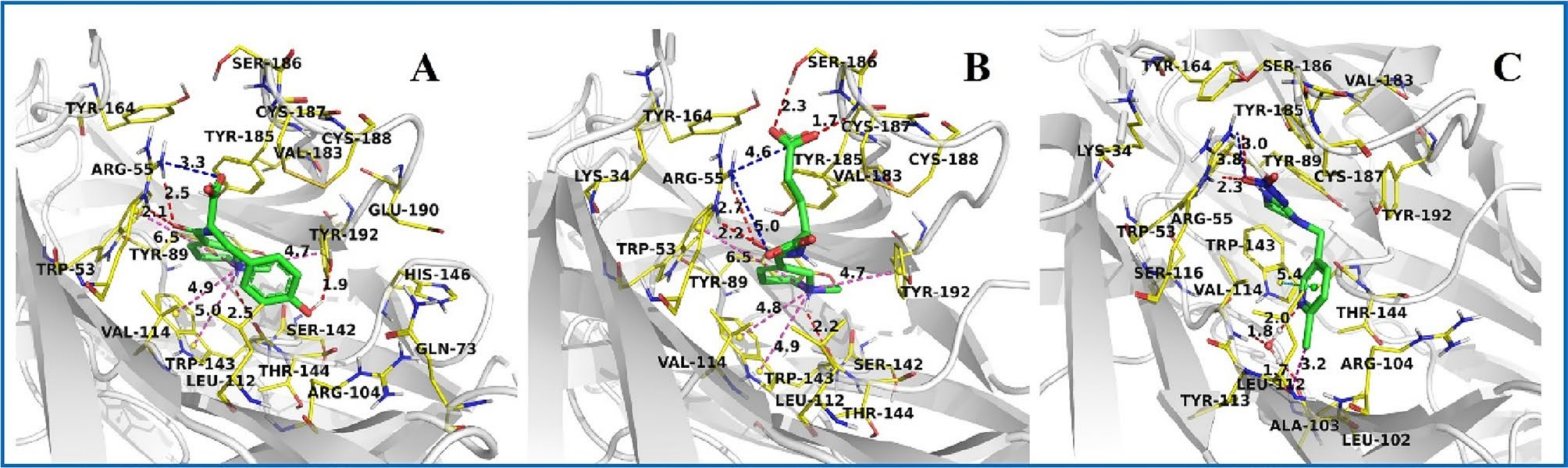
Molecular docking results of different chemicals with AChBP. (**A**) **3h**, (**B**) **3i** and (**C**) Imidacloprid. The small molecules are shown in green sticks, while the residues within 5 Å of the ligand are shown in yellow lines. The hydrogen bond, cation-π, salt bridge and halogen bond are represented by red dash, light-magenta dash, blue dash and purple dash.

Figure 2 illustrates the binding modes of target compounds **3h** and **3i** to AChBP, revealing similarities between them. Both compounds establish hydrogen bonds with TRP-143 and engage in four cation–π interactions with TRP-143, TRP-53, and TYR-192, in addition to forming two hydrogen bonds with ARG-55. Specifically, compound **3i** formed two hydrogen bonds with SER-186 and CYS-187 and two salt bridges with ARG-55, whereas compound **3h** formed only one salt bridge interaction with ARG-55 and an additional hydrogen bond with TYR-192. This correlates with the observed stronger biological effects of **3i** compared to **3h**. The reference molecule, imidacloprid (IMI) formed two hydrogen bonds and a salt bridge with ARG-55. In particular, imidacloprid shared a halogen bond with LEU112 and a water bridge with LEU102 and VAL114. These interactions between IMI molecules and AChBPs may be vital factors for the significant insecticidal activity of IMI.

### Conclusions

In summary, this research successfully designed and synthesised a series of novel arecoline derivatives containing amino acid functional groups. The insecticidal and fungicidal efficacy of these compounds were assessed. Compounds **3h** and **3i** exhibited higher insecticidal activities against *A. craccivora* (LC_50_: 394.66 and 275.28 mg/L) and *T. cinnabarinus* (LC_50_: 208.01 and 257.46 mg/L) as arecoline (LC_50_: 634.19 and 315.35 mg/L). Furthermore, compounds **3h** and **3i** showed excellent inhibitory activities against *C. gloeosporioides* (54% and 48%) and *B. cinerea* (70% and 64%). Molecular docking analyses suggest that **3h** and **3i** likely target neuronal nicotinic acetylcholine receptors. This study contributes valuable insights and introduces promising candidates for the development of new arecoline-based insecticides and fungicides. Future work will explore the acute toxicity and detailed mechanisms of action of these compounds in our laboratory.

## Materials and methods

### Chemical and instruments

All reagents and solvents were sourced from commercial suppliers and utilised as received, without further purification. Arecoline (98% purity) was purchased from Shanghai Maclean Biochemical Technology Co., Ltd. 2-(7-Azabenzotriazol-1-yl)-*N*, *N*, *N*ʹ, *N*ʹ-tetramethyluronium hexafluorophosphate (99% purity) (HATU), *N*, *N*-Diisopropylethylamine (99% purity) (DIEA) and amino acids were from Shanghai Aladdin Reagent Co Ltd. Solid reagents such as sodium hydroxide and sodium carbonate are analytically pure grade, produced by Tianjin City Zhiyuan Chemical Reagent Co., Ltd. Methanol, ethanol, anhydrous ether, dichloromethane and other liquid reagents are analytically pure grade, are purchased from Guangzhou Chemical Reagent Factory. Deionized water was obtained from laboratory preparation.

NMR spectra were obtained using a Bruker Advance spectrometer operating at 400 MHz, using MeOD with TMS as the internal standard. Melting points were determined using a Buchi melting point (B540) apparatus and were reported as observed. High-resolution mass spectra were obtained on a Triple TOF 5600 mass spectrometer (AB SCIEX, USA).

### Synthesis

#### Synthesis of compound 1

Compound **1** was synthesised as previously described^32^. Initially, arecoline hydrobromide (5.90 g, 25 mmol) was dissolved in 30 mL of deionised water. The pH was adjusted to 10–11 using 30% Na_2_CO_3_, and the solution was stirred at room temperature for 1 h. Then, the mixture was extracted with DCM (5 × 10 mL). The DCM extracts were then concentrated under vacuum at 40 ℃. The residue was dissolved in 30 mL of water and refluxed overnight after heating. Finally, water was removed under vacuum and the crude product was purified by recrystallisation using a water-acetonitrile mixture, yielding compound **1**.

Compound **1**, white solid, yield 92%, mp. 225–226 °C; ^**1**^**H-NMR** (400 MHz, D_2_O) *δ* 6.70–6.72 (m, 1H), 3.98–4.02 (d, *J* = 16.0, 1H), 3.59–3.64 (d, *J* = 20.0, 1H), 3.44–3.46 (m, 1H), 3.04–3.11 (m, 1H), 2.89 (s, 3H), 2.41–2.60 (m, 2H). ^**13**^**C NMR** (400 MHz, D_2_O) *δ* 171.8, 131.9, 128.5, 52.1, 49.7, 42.2, 22.3.

#### General procedure for compounds 2a–2l

The intermediate **2a–2l** were synthesised following the methodology outlined in our previous study, as detailed in Scheme B^31^. In briefly, 16 mL (400 mmol) of methanol was added to a 50 mL round-bottom flask and placed in ice-salt bath conditions. Keep the temperature at − 5 °C, slowly add 1.9 mL (26 mmol) SOCl_2_ dropwise with a constant pressure dropping funnel, and control the addition to be completed within 30 min. After stirring at − 5 °C for 1 h, the system was allowed to stand at room temperature and 1.5 g (20 mmol) of l-alanine was added. After stirring at room temperature for 1 h, heat at 70 °C for 2 h at reflux. After the reaction was finished, the unreacted methanol and SOCl_2_ were removed by pressure reduction and concentration at 50 °C to obtain a crude product. Recrystallize with methanol (a little)-anhydrous ether (excess) in ice water bath, then an amount of white lump crystal was precipitated, the crystals were washed with anhydrous ether for 2–3 times, and the pure product compound **2d** was obtained by vacuum drying. The synthetic procedures of compounds **2a–2c** and **2e–2i** were prepared similarly to **2d**.

#### General procedure for target compounds 3a–3l

In a 50 mL flask, compound 1 (169 mg, 1.2 mmol), HATU (532 mg, 1.4 mmol), DIEA (0.8 mL), and DCM (10 mL) were combined and cooled to − 5 °C in an ice bath. The mixture was stirred at − 5 °C for 1 h before adding compound **2a** (255 mg, 1 mmol), continuing the stirring at − 5 °C for 1 h. Completion of reaction was monitored by TLC. The mixture was then washed with deionised water (5 mL × 3), and the organic phase was dried under vacuum to yield a pale-yellow oil. This crude product was used directly in the next step without purification.

The crude oil was dissolved in a 3 mL mixture of water and tetrahydrofuran (H_2_O: THF = 1:2), to which NaOH (120 mg, 3 mmol) was added. The solution was stirred thoroughly for 2 h at room temperature. It was then transferred to a 60 mL separatory funnel and extracted with 2 mL of DCM. After layer separation, the organic layer was collected and concentrated under reduced pressure to yield crude product **3a**. This product was further purified by recrystallisation (water/acetone, 1:15 v/v) to obtain the desired compound **3a**. The synthesis of compounds **3b–3l** followed a similar procedure.

##### Compound 3a

Sodium (1-methyl-1, 2, 5, 6-tetrahydropyridine-3-carbonyl) tryptophanate. Yellow powder, yield 35.4%, mp.107–108 °C; ^1^H NMR (400 MHz, MeOD) *δ* 7.50 (dt, *J* = 8.0, 1.2 Hz, 1H), 7.25 (dt, *J* = 8.0, 1.2 Hz, 1H), 7.04 (s, 1H), 7.01–6.92 (m, 2H), 6.40 (m, 1H), 4.58 (m, 1H), 3.39–3.20 (m, 2H), 3.08–2.86 (m, 2H), 2.48–2.40 (m, 2H), 2.28 (s, 3H), 2.24–2.19 (m, 2H). ^13^C NMR (400 MHz, MeOD) *δ* 177.3, 166.6, 136.5, 131.6, 130.5, 128.2, 123.1, 120.7, 118.3, 118.1, 110.7, 110.5, 55.8, 52.4, 50.3, 44.2, 27.4, 25.1. HRMS-ESI (m/z): calcd for C_18_H_20_N_3_NaO_3_ [M+H]^+^ 350.1475, found, 350.1482.

##### Compound 3b

Sodium (1-methyl-1,2,5,6-tetrahydropyridine-3-carbonyl) leucinate. Yellow oil, yield 36%; ^1^H NMR (400 MHz, MeOD) *δ* 6.63 (m, 1H), 4.38 (m, 1H), 3.23–3.08 (m, 2H), 2.55–2.49 (m, 2H), 2.37 (s, 3H), 2.35–2.30 (m, 2H), 1.67–1.60 (m, 2H), 1.59–1.55 (m, 1H), 0.92 (d, *J* = 1.6 Hz, 3H), 0.90 (d, *J* = 1.2 Hz, 3H). ^13^C NMR (400 MHz, MeOD) *δ* 178.8, 167.0, 131.8, 130.3, 53.5, 52.7, 50.4, 44.3, 42.0, 25.3, 24.9, 22.4, 21.0. HRMS-ESI (m/z): calcd for C_13_H_21_N_2_NaO_3_ [M+H]^+^ 277.1522, found, 277.1530.

##### Compound 3c

Methyl (1-methyl-1,2,5,6-tetrahydropyridine-3-carbonyl) valinate. Yellow oil, yield 38%; ^1^H NMR (400 MHz, MeOD) *δ* 6.64 (m, 1H), 4.20 (d, *J* = 5.6 Hz, 1H), 3.29–3.07 (m, 2H), 2.59–2.50 (m, 2H), 2.38 (s, 3H), 2.35–2.28 (m, 2H), 2.16–2.07 (m, 1H), 0.93 (d, *J* = 7.2Hz, 3H), 0.88 (d, *J* = 6.8 Hz, 3H). ^13^C NMR (400 MHz, MeOD) *δ* 177.3, 167.1, 131.8, 130.4, 60.0, 52.7, 50.4, 44.3, 31.5, 25.3, 18.8, 17.2. HRMS-ESI (m/z): calcd for C_13_H_22_N_2_O_3_ [M+H]^+^ 255.1703 found, 255.1712.

##### Compound 3d

Sodium (1-methyl-1, 2, 5, 6-tetrahydropyridine-3-carbonyl) alaninate. Yellow oil, yield 30%; ^1^H NMR (400 MHz, MeOD) *δ* 6.69 (m, 1H), 4.27 (q, *J* = 6.0 Hz, 1H), 3.20 (m, 2H), 2.57 (m, 2H), 2.42 (s, 3H), 2.39 (m, 2H). ^13^C NMR (400 MHz, MeOD) *δ* 178.3, 166.4, 131.7, 130.3, 52.5, 50.5, 50.4, 44.2, 25.2, 18.0. HRMS-ESI (m/z): calcd for C_10_H_15_N_2_NaO_3_ [M+H]^+^ 235.1053 found, 235.1062.

##### Compound 3e

Sodium (1-methyl-1, 2, 5, 6-tetrahydropyridine-3-carbonyl) methioninate. Yellow oil, yield 33%; ^1^H NMR (400 MHz, MeOD) *δ* 6.70 (m, 1H), 4.38 (dd, *J* = 6.0, 4.0 Hz, 1H), 3.22 (m, 2H), 2.58 (m, 2H), 2.52 (m, 2H), 2.42 (s, 3H), 2.39 (m, 2H), 2.15 (m, 2H), 2.08 (s, 3H), 2.00 (m, 2H). ^13^C NMR (400 MHz, MeOD) *δ* 176.9, 166.8, 131.6, 130.6, 54.3, 50.4, 50.1, 44.2, 32.4, 30.0, 25.2, 13.9. HRMS-ESI (m/z): calcd for C_12_H_19_N_2_NaO_3_S [M+H]^+^ 295.1086, found, 295.1092.

##### Compound 3f

Sodium (1-methyl-1,2,5,6-tetrahydropyridine-3-carbonyl) phenylalaninate. Yellow oil, yield 40%; ^1^H NMR (400 MHz, MeOD) *δ* 7.25–7.15 (m, 5H), 6.54 (m, 1H), 4.56 (dd, *J* = 5.6, 4.0 Hz, 1H), 3.25 (dd, *J* = 10.8, 4.0 Hz, 2H), 3.16 (dd, *J* = 12.8, 1.2 Hz, 2H), 3.05 (m, 2H), 2.25 (m, 2H), 2.38 (s, 3H), 2.33 (m, 2H). ^13^C NMR (400 MHz, MeOD) *δ* 176.6, 166.6, 138.0, 131.7, 130.3, 129.2, 127.7, 125.9, 55.7, 52.5, 50.3, 44.2, 37.6, 25.1. HRMS-ESI (m/z): calcd for C_16_H_19_N_2_NaO_3_ [M+H]^+^ 311.1366, found, 311.1375.

##### Compound 3g

Sodium (1-methyl-1, 2, 5, 6-tetrahydropyridine-3-carbonyl) aspartate. Yellow solid, yield 35%, mp. 235–236 °C; ^1^H NMR (400 MHz, MeOD) *δ* 6.72 (m, 1H), 4.53(t, *J* = 4.0 Hz, 1H), 3.22(dd, *J* = 24.4, 12.8 Hz, 2H), 2.69 (m, 2H), 2.56 (m, 2H), 2.41(s, 3H), 2.38 (m, 2H). ^13^C NMR (400 MHz, MeOD) *δ* 178.8, 178.3, 131.6, 130.6, 52.6(2C), 50.4, 44.3, 40.5, 25.2. HRMS-ESI (m/z): calcd for C_11_H_14_N_2_Na_2_O_5_ [M+H]^+^ 301.0770, found, 301.0779.

##### Compound 3h

Sodium 3-(1-methyl-1, 2, 5, 6-tetrahydropyridine-3-carboxamido)-3-(4-oxidophenyl) propanoate. Yellow solid, yield 40%, mp. 127–129 °C; ^1^H NMR (400 MHz, MeOD) *δ* 6.89–6.85 (d, *J* = 4.0 Hz, 2H), 6.54–6.52(d, *J* = 8.0 Hz, 2H), 6.52 (m, 1H), 4.43(t, *J* = 4.0 Hz, 1H), 3.20–3.17(m, 2H), 3.07–3.03 (m, 2H), 2.57–2.50(m, 2H), 2.38(s, 3H), 2.33(m, 2H). ^13^C NMR (400 MHz, MeOD) *δ* 177.7, 166.7, 165.2, 131.8, 130.2, 129.7, 122.4, 118.2, 56.3, 52.5, 50.4, 44.2, 36.8, 25.1. HRMS-ESI (m/z): calcd for C_16_H_18_N_2_Na_2_O_4_ [M+H]^+^ 349.1134, found, 349.1149.

##### Compound 3i

Sodium (1-methyl-1, 2, 5, 6-tetrahydropyridine-3-carbonyl) glutamate. Yellow solid, yield 42%, mp. 268–270 °C; ^1^H NMR (400 MHz, MeOD) *δ* 6.73 (m, 1H), 4.26 (m, 1H), 3.27–3.18 (dd, *J* = 24, 16 Hz, 2H), 2.58 (m, 2H), 2.42 (s, 3H), 2.39 (m, 2H), 2.13 (m, 2H), 2.03 (m, 2H). ^13^C NMR (400 MHz, MeOD) *δ* 181.0, 177.9, 166.9, 131.7, 130.2, 55.5, 52.6, 50.4, 44.2, 34.2, 29.0, 25.2. HRMS-ESI (m/z): calcd for C_12_H_16_N_2_Na_2_O_5_ [M+H]^+^ 315.0927, found, 315.0938.

### Statistical analysis

Statistical analysis was performed using GraphPad Prism (ver. 8.3; GraphPad Software, San Diego, CA, USA). The Duncan’s multiple range test (p < 0.05) was used for data analysis, and the experimental data were expressed as the mean ± standard deviation (SD).

### Biological assay

Detailed bioassay procedures for antifungal and insecticidal activities are referenced from published literature^11,33,34^. The relevant insect sources and strains employed in the experiment were sourced from the Institute of Plant and Environmental Protection of the Chinese Academy of Tropical Agricultural Sciences and the Institute of Tropical Biotechnology, respectively. Each bioassay is repeated at least 3 times to meet statistical requirements.

#### Insecticidal activities

Prepare a stock solution of each tested compound with methanol at a concentration of 3200 mg/L, and then dilute it to the desired concentration (50,100, 200, 400, 800, 1600 and 3200 mg/L) with deionized water. Deionized water (or methanol) and Imidacloprid (concentrations ranging from 2 to 32 mg/L) were used as blank and positive controls, respectively. Leaf-dip method was executed. Cut a leaf disc measuring 5 cm × 3 cm from fresh cowpea leaves, and then immerse it in the test solution for 10 s. After air-drying, the petioles were moisturized with skimmed cotton dipped in water, and the processed leaf discs were placed separately in a constant temperature chamber and inoculated with 30 aphids (wingless adults and larger wingless nymphs of aphids). During the examination, the aphids were gently flicked with a brush, and if they could not stand up after falling down, they were considered dead. Experimental conditions: temperature (25 ± 1) °C, relative humidity of about 75%, light cycle L:D = 16:8. Mortality rates were recorded over 48 h and toxicity of the corresponding compounds were determined by further determination of median lethal concentrations with mortality exceeding 50% (LC_50_).

The insecticidal activity of title compounds against *T. cinnabarinus* was tested according to our previously reported procedures^35^.

#### Antifungal activities

The antifungal activity of the test compound against the selected five phytopathogenic fungi was evaluated using an agar well diffusion method^36^. Briefly, dissolve the test compound in an appropriate amount of methanol, and then use a solution containing prepare a 0.1% TW-80 aqueous solution to obtain a stock solution with a concentration of 500 mg/L. One milliliters of stock solution were added to the sterilized PDA medium. The final concentrations were 50 mg/L. Subsequently, a Foc TR4 disc (4 mm) was placed on the PDA plate and incubated at 24 ± 1 °C for 48 h. sterilized water treatment was used as a control. The mycelial growth diameter was measured and the percentage inhibition was calculated.

$$Percentage \, inhibition \, \left( \% \right) \, = \, \left( {averaged \, diameter \, of \, mycelia \, in \, blank \, controls \, {-} \, averaged \, diameter \, of \, mycelia \, in \, medicated \, tablets} \right) \, / \, averaged \, diameter \, of \, mycelia \, in \, blank \, controls.$$

### Docking simulation

Molecular docking analysis was performed using *Schrodinger Suite 2023.4*. The structure of *Lymnaea stagnalis* AChBP with a Gln55Arg mutation (PDB Entry: 7PDR) was selected as the receptor in our docking^37,38^. Both the receptor and the small molecules underwent preparation processes using the *Protein Preparation Workflow* and *LigPrep* within the OPLS4 force field, separately. The receptor grid was then generated using *Receptor Grid Generation*. Finally, our small molecules were docked into the receptor at the reference pocket with extra precision using the default parameters by *Ligand Docking*.

### Supplementary Information

Supplementary Figures.

## Data Availability

The data that support the findings of this study are available in the Supplementary Material of this article.
