# Supplementary material for: Design, synthesis, and evaluation of novel arecoline-linked amino acid derivatives for insecticidal and antifungal activities
Source: Sci Rep. 2024 Apr 24;14:9392. doi: 10.1038/s41598-024-60053-2 (PMC11043403; doi:10.1038/s41598-024-60053-2)
Supplement: Supplementary file 1 — Supplementary Figures. [file 41598_2024_60053_MOESM1_ESM.docx]

**Supporting Information**

**Design, Synthesis, and Evaluation of Novel Arecoline-Linked Amino Acid Derivatives for Insecticidal and Antifungal Activities**

Chaohai Pang ^1,^ *^, +^, Yuan Xu^2, +^, Xionghui Ma^1,^ *, Shuhuai Li^1,^ *, Shengfu Zhou^3^, Hai Tian^1^, Mingyue Wang^1^, Bingjun Han^1^

^1^ Analysis and Test Center, Chinese Academy of Tropical Agricultural Sciences, Hainan Provincial Key Laboratory of Quality and Safety for Tropical Fruits and Vegetables, Key Laboratory of Quality and Safety Control of Subtropical Fruits and Vegetables, Ministry of Agriculture and Rural Affairs, Haikou, 571101, China.

^2^ Key Laboratory of Tropical Translational Medicine of Ministry of Education, Hainan Provincial Key Laboratory for Research and Development of Tropical Herbs, School of Pharmacy, Hainan Medical University, Haikou 571199, China.

^3^ BayRay Innovation Center, Shenzhen Bay Laboratory, Shenzhen, 518000, China.

*Corresponding author’s e-mail: [18389859589@163.com](mailto:18389859589@163.com) (Chaohai Pang); [maxionghui@foxmail.com](mailto:maxionghui@foxmail.com) (Xionghui Ma); [happylishuhuai@163.com](mailto:happylishuhuai@163.com) (Shuhuai Li).

**^+^**These authors contributed equally: Chaohai Pang and Yuan Xu.

Table of Contents

[1.^1^H NMR spectrum of compound 3h (before/after recrystallisation) 2](#_Toc163059536)

[2. NMR spectrum for target compounds 3a~3i 3](#_Toc163059537)

[3. HRMS - ESI (m/z) spectrum for target compounds 3a~3i 12](#_Toc163059538)

# 1.^1^H NMR spectrum of compound 3h (before/after recrystallisation)


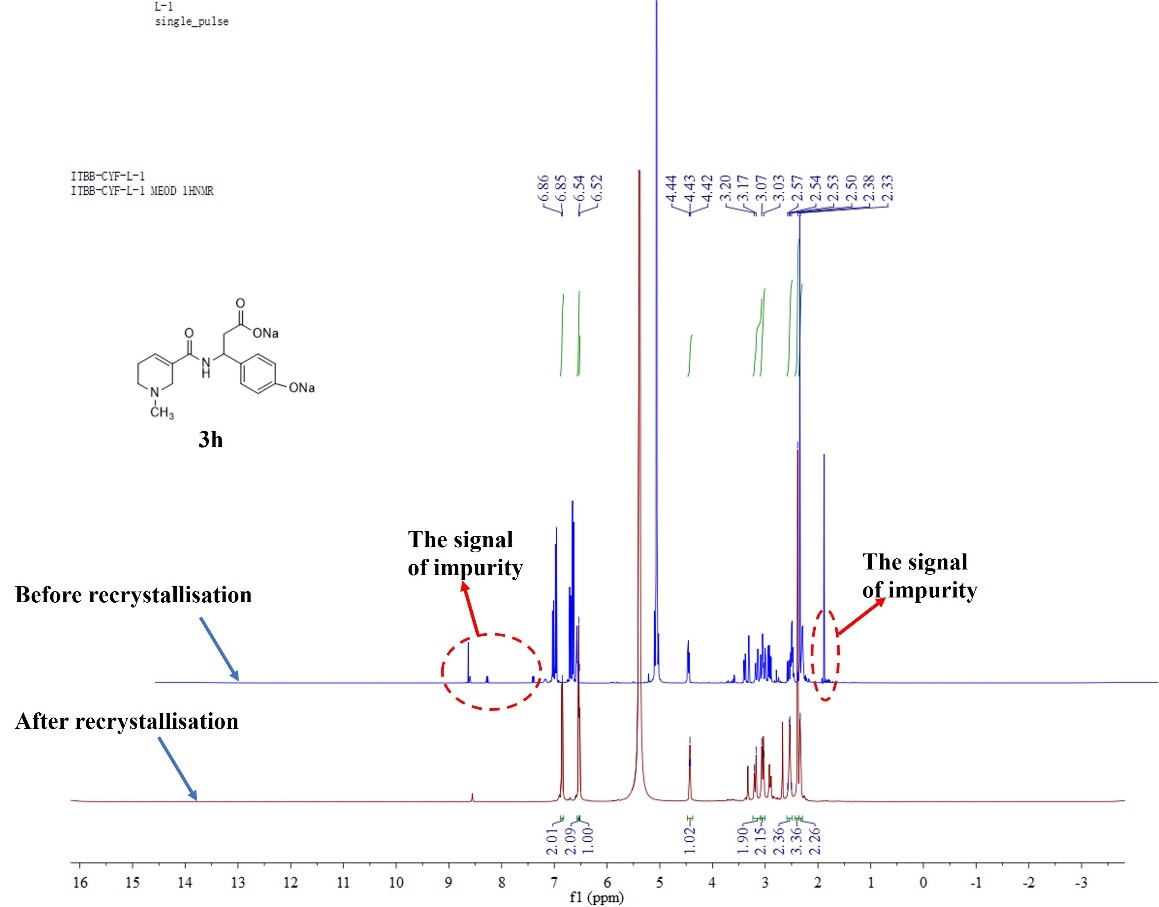
 Supplementary Fig.S1 Comparison of ^1^H NMR spectrum for compound 3h (before/after recrystallisation)

# 2. NMR spectrum for target compounds 3a~3i


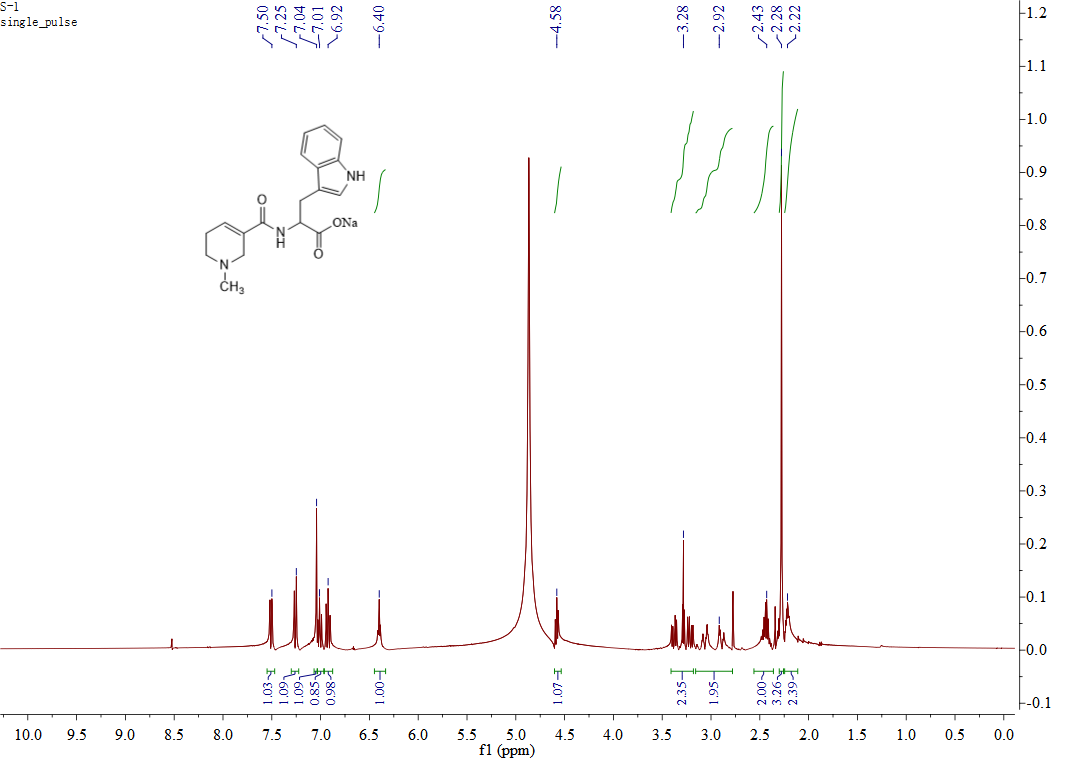


Supplementary Fig. S2 ^1^H NMR spectrum for compound 3a-A


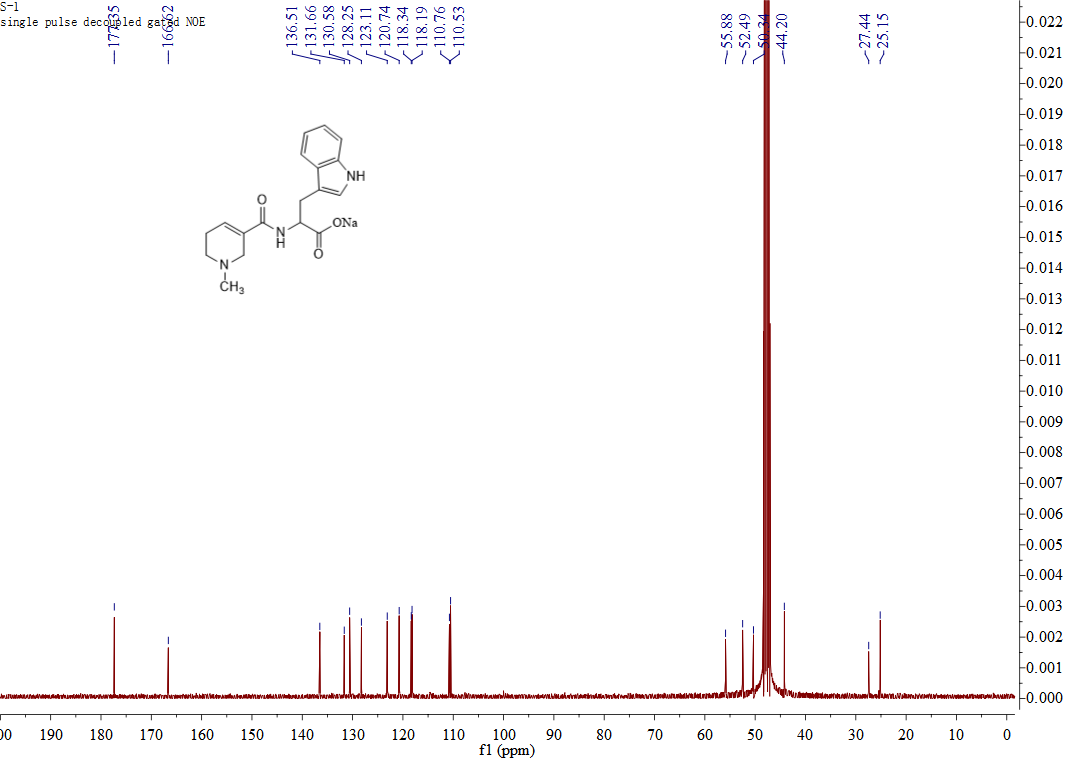


Supplementary Fig.S3 ^13^C-NMR spectrum for compound 3a-B


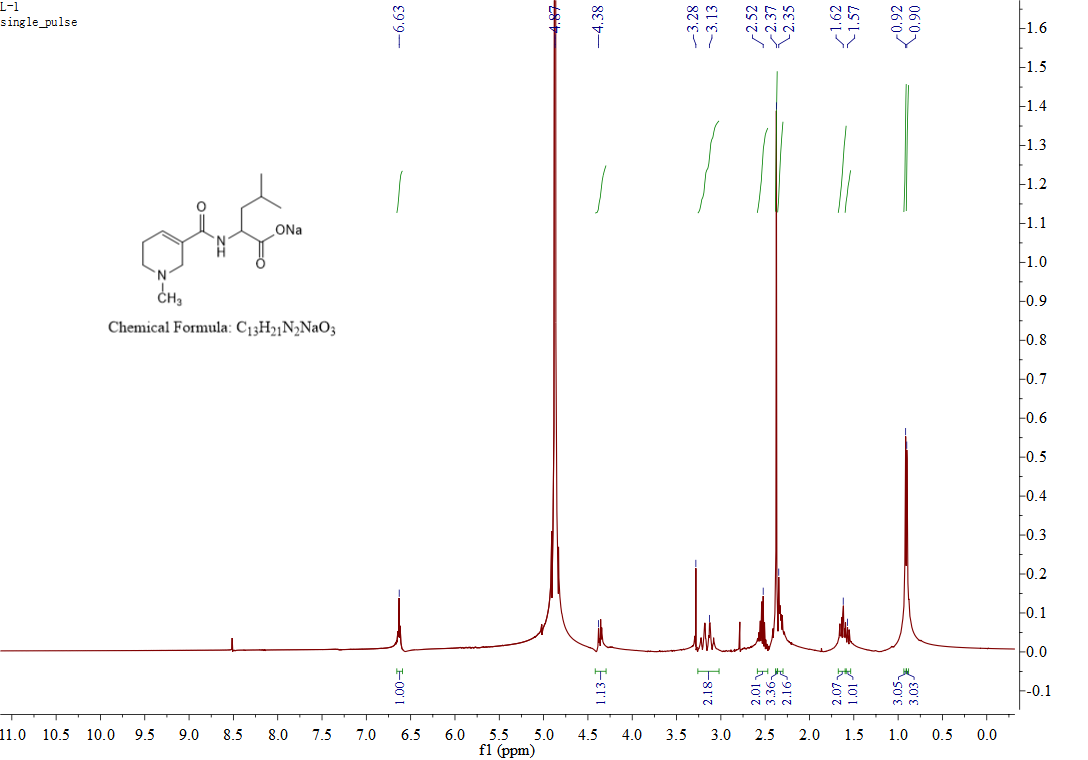


Supplementary Fig.S4 ^1^H NMR spectrum for compound 3b-A


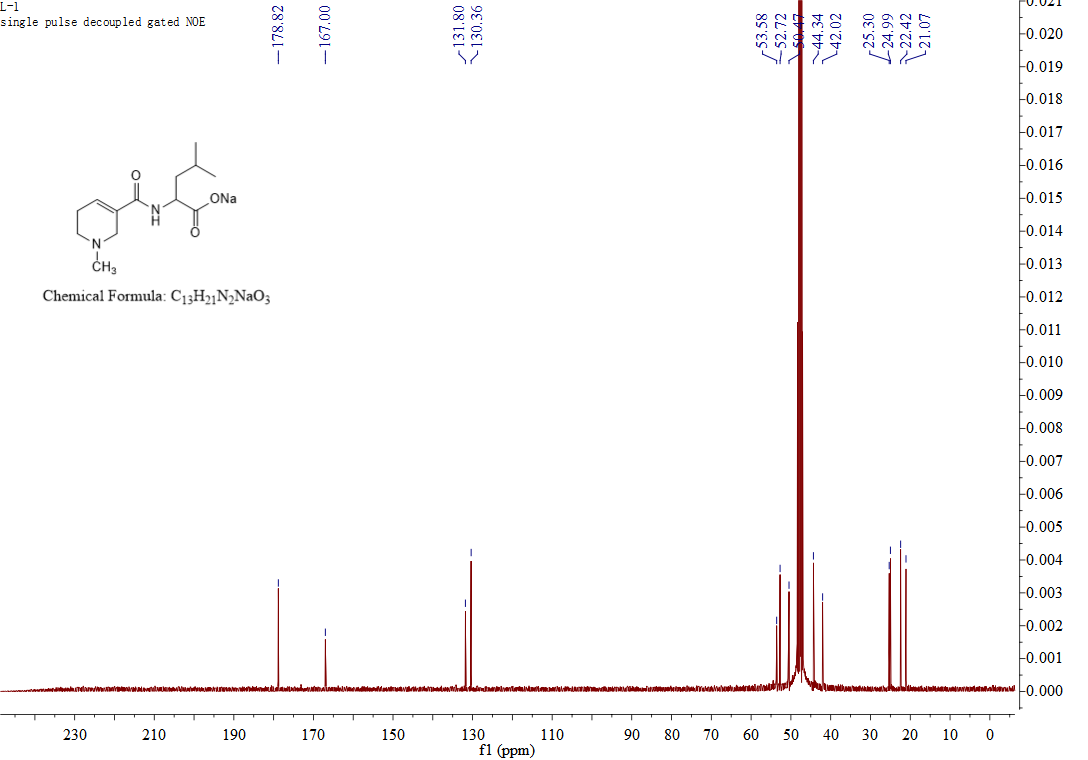


Supplementary Fig.S5 ^13^C-NMR spectrum for compound 3b-B


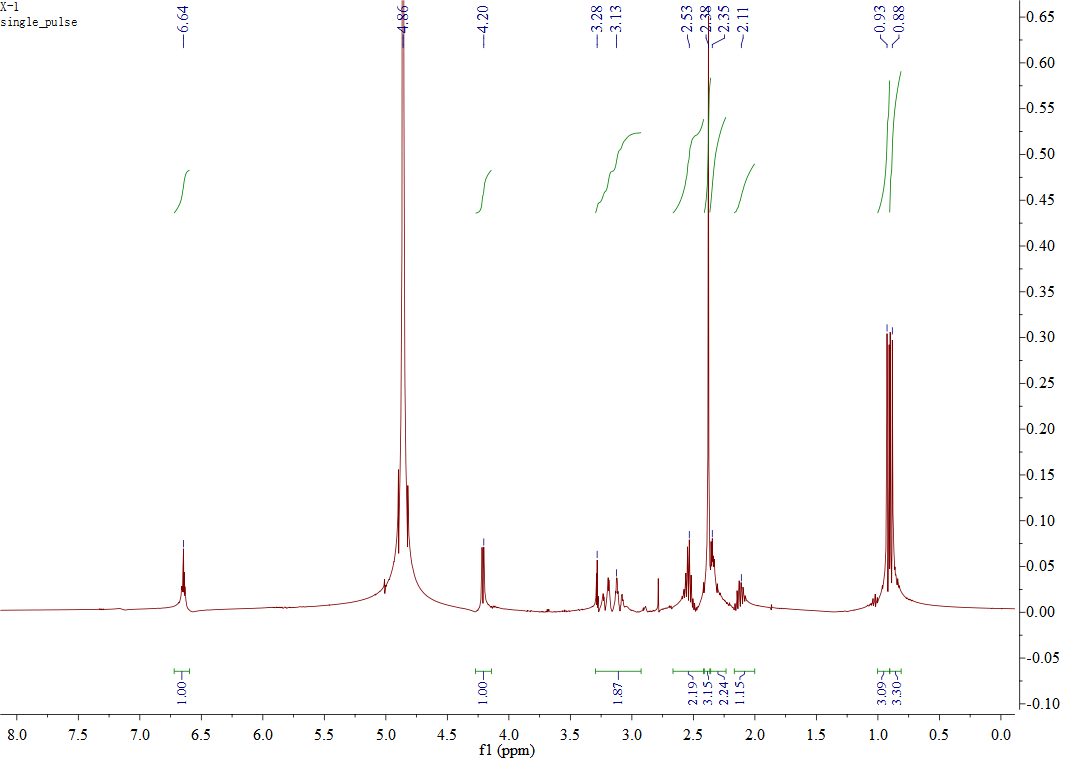


Supplementary Fig.S6 ^1^H NMR spectrum for compound 3c-A


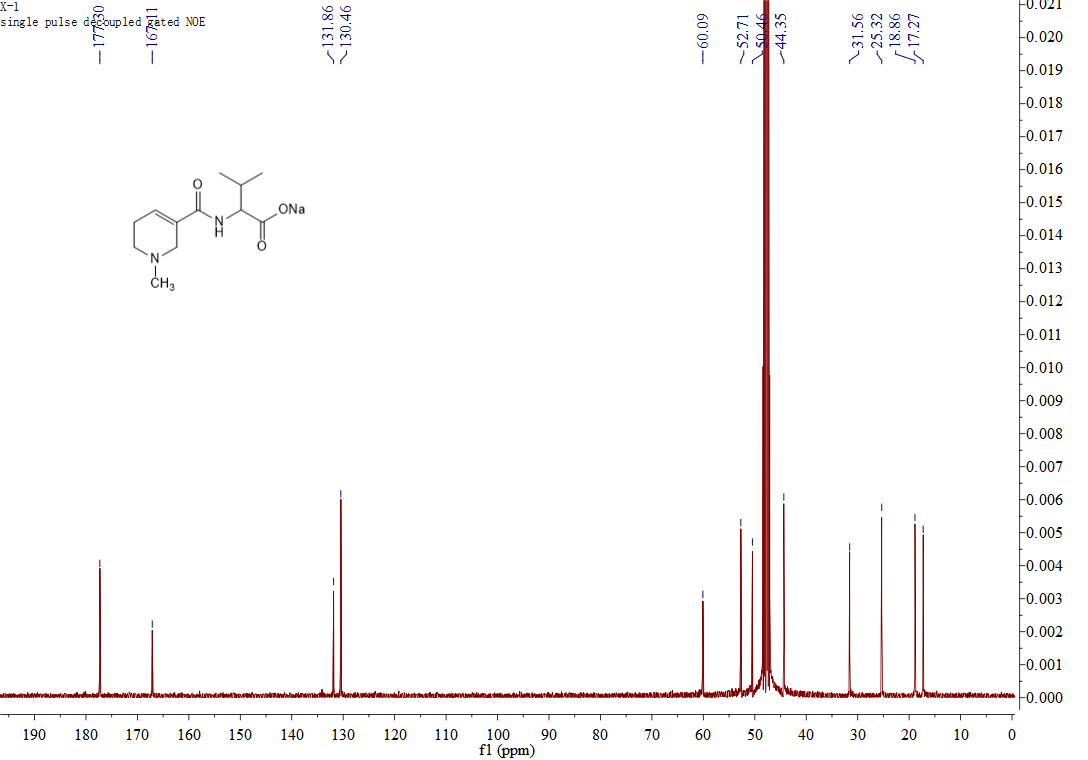


Supplementary Fig.S7 ^13^C-NMR spectrum for compound 3c-B


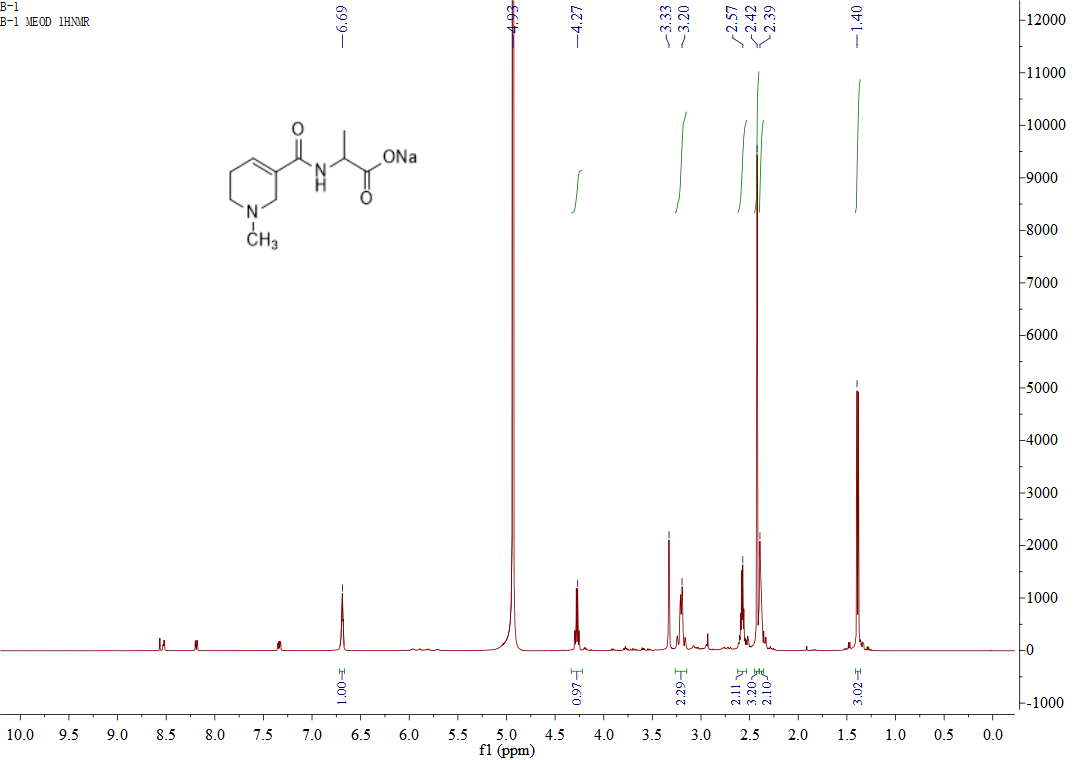


Supplementary Fig.S8 ^1^H NMR spectrum for compound 3d-A


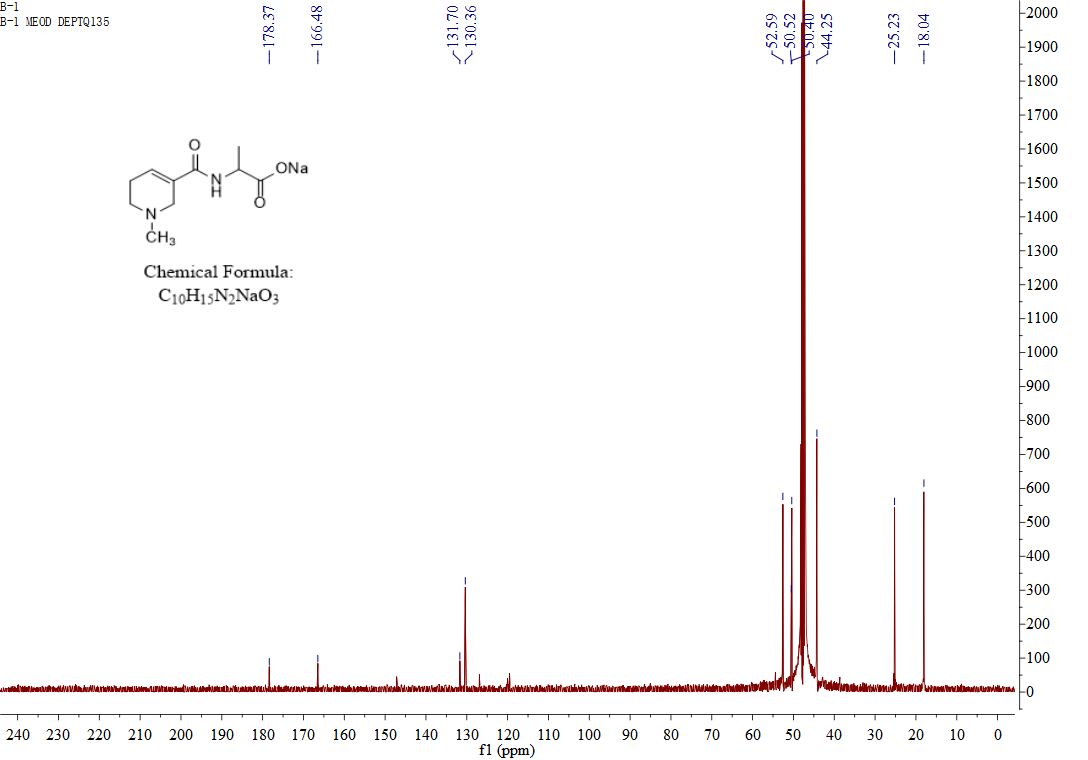


Supplementary Fig.S9 ^13^C-NMR spectrum for compound 3d-B


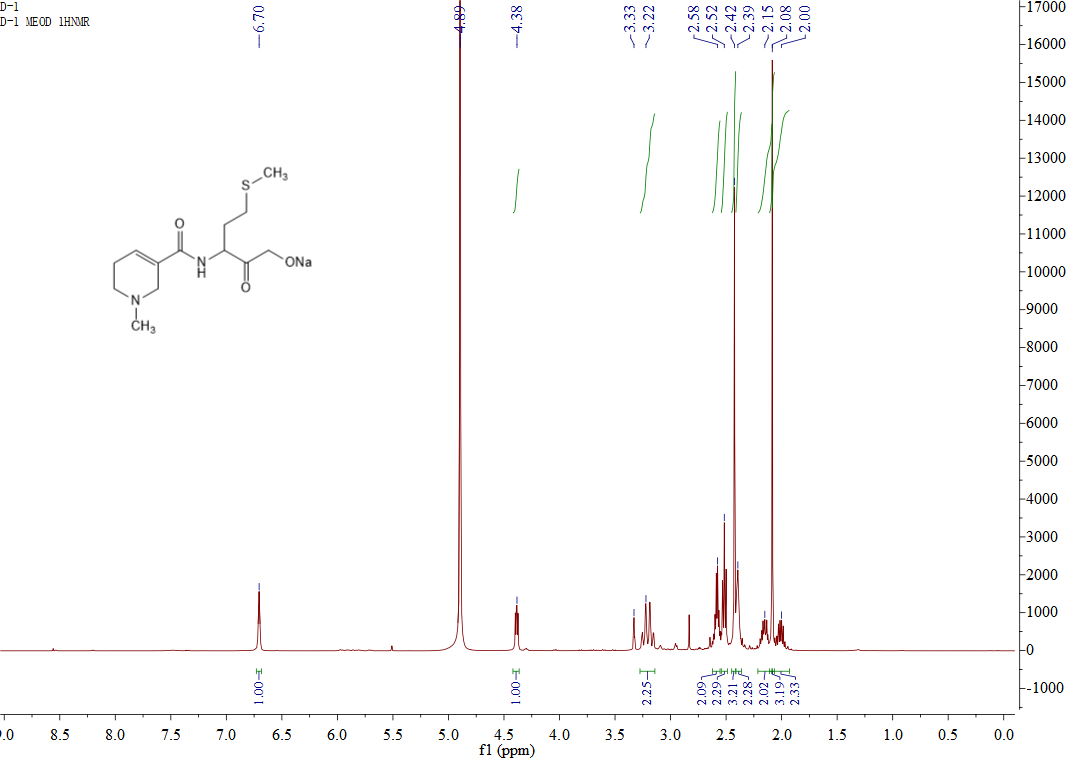


Supplementary Fig.S10 ^1^H NMR spectrum for compound 3e-A


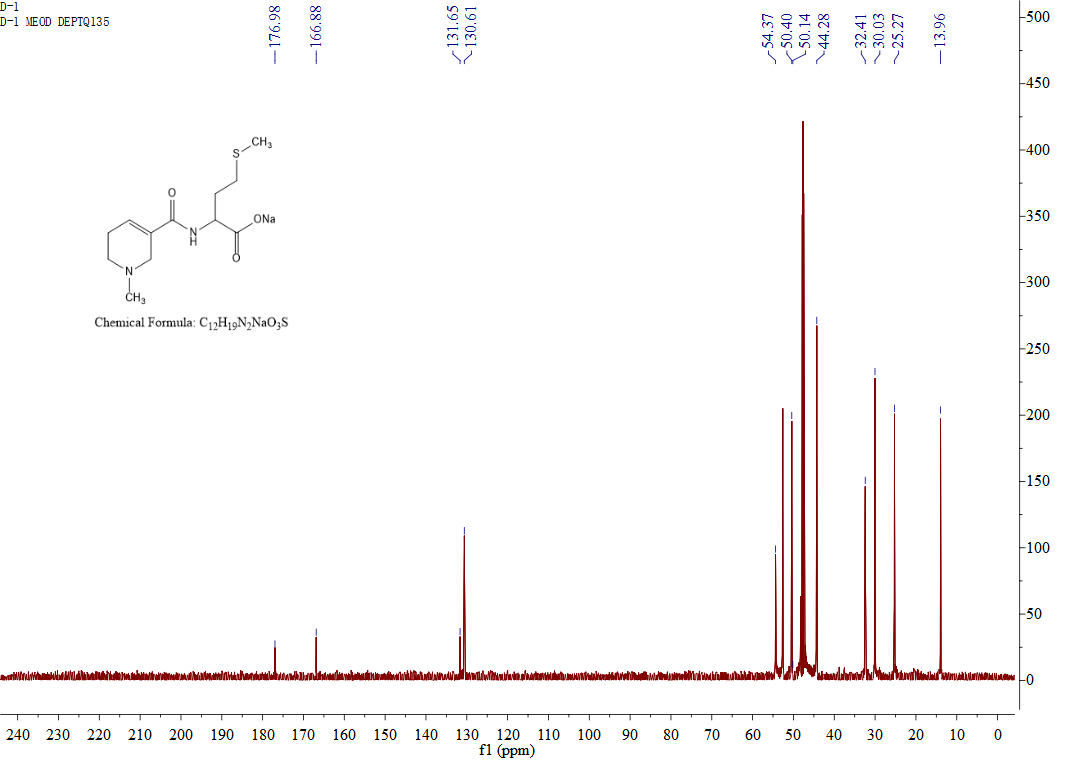


Supplementary Fig.S11 ^13^C-NMR spectrum for compound 3e-B


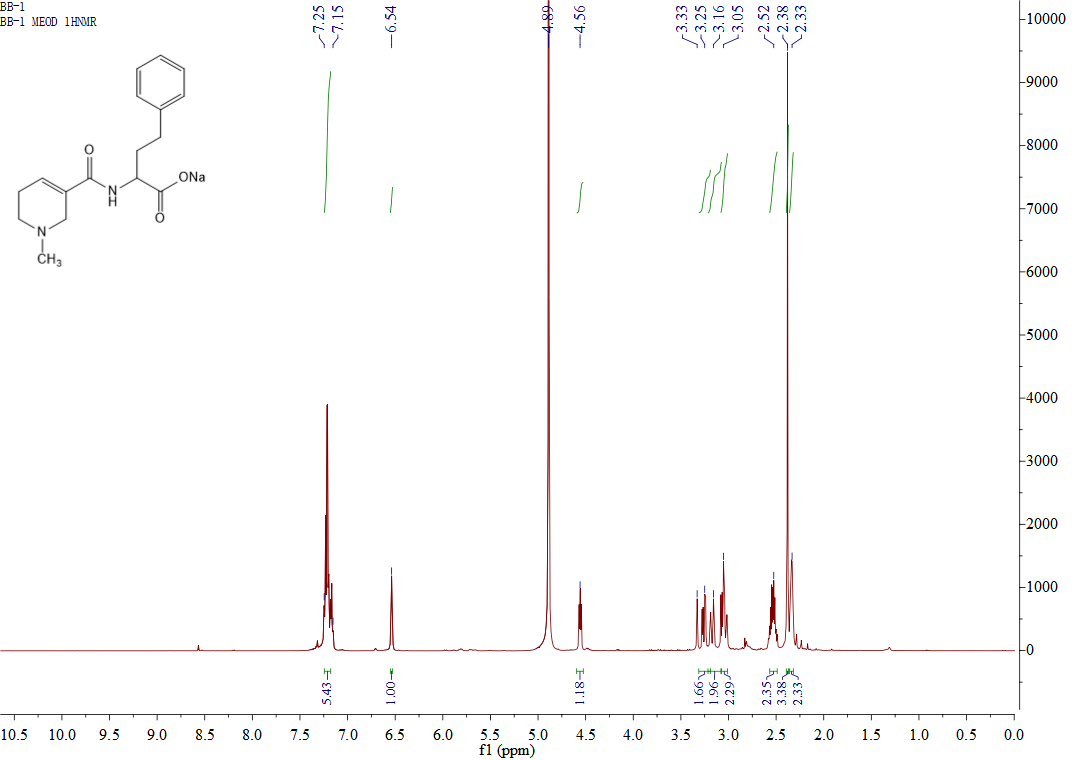


Supplementary Fig.S12 ^1^H NMR spectrum for compound 3f-A


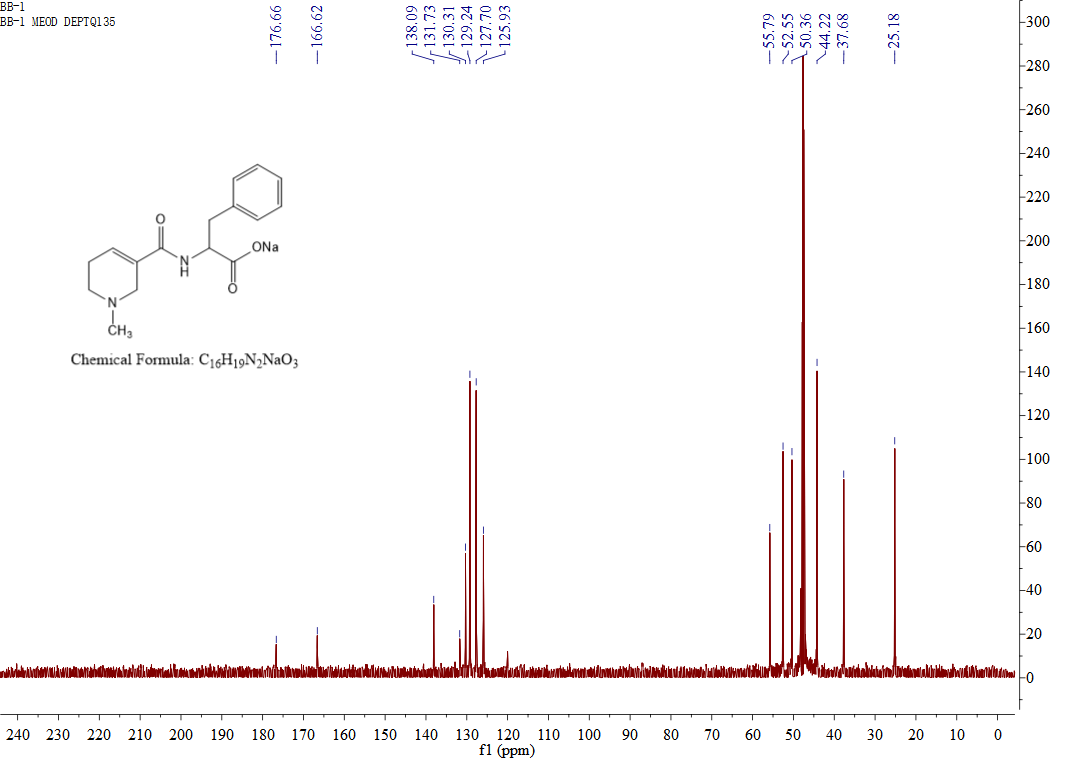


Supplementary Fig.S13 ^13^C-NMR spectrum for compound 3f-B


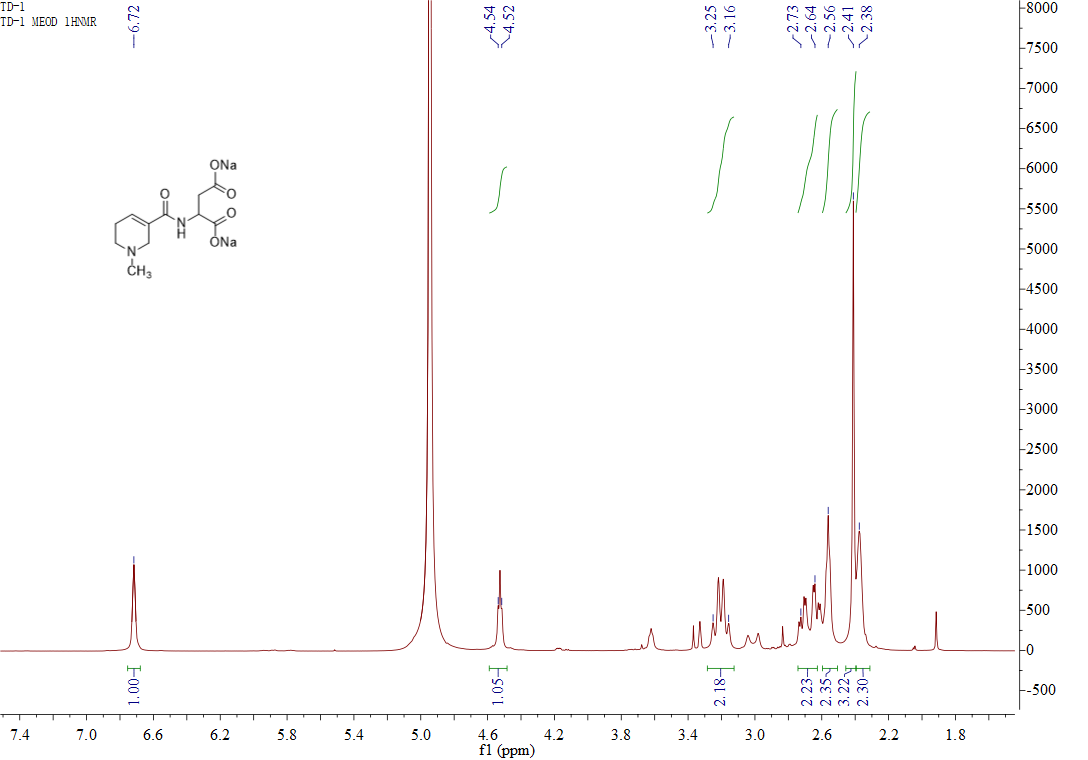


Supplementary Fig.S14 ^1^H NMR spectrum for compound 3g-A


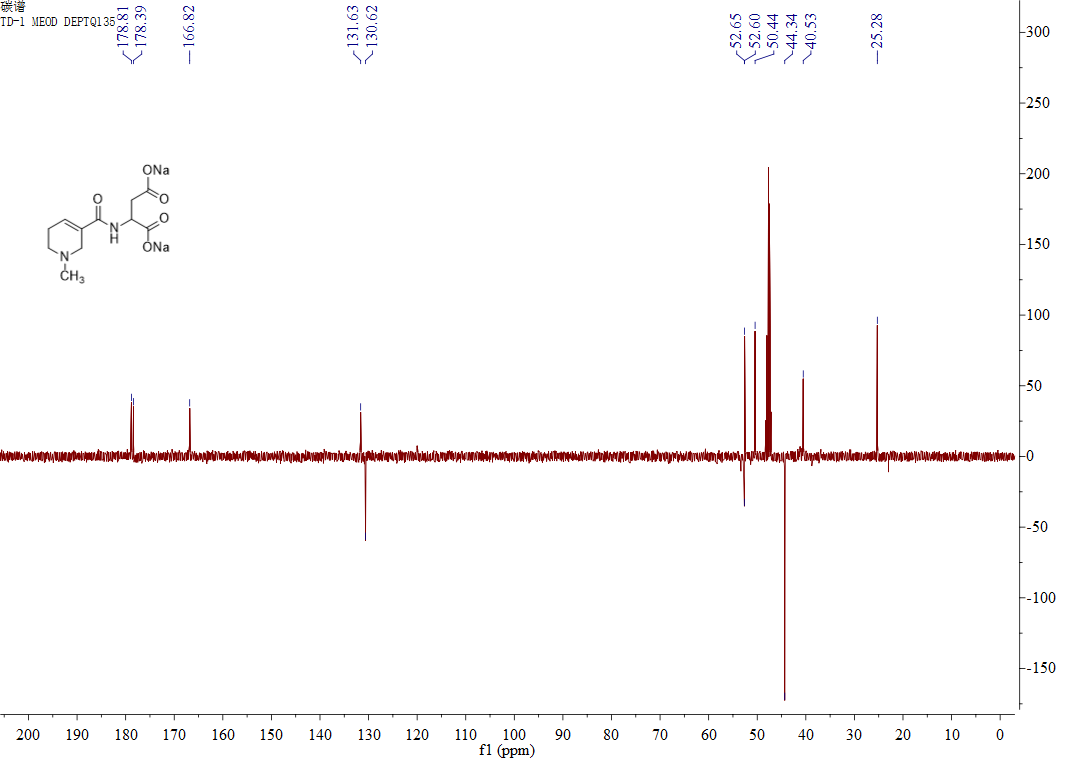


Supplementary Fig.S15 ^13^C-NMR spectrum for compound 3g-B


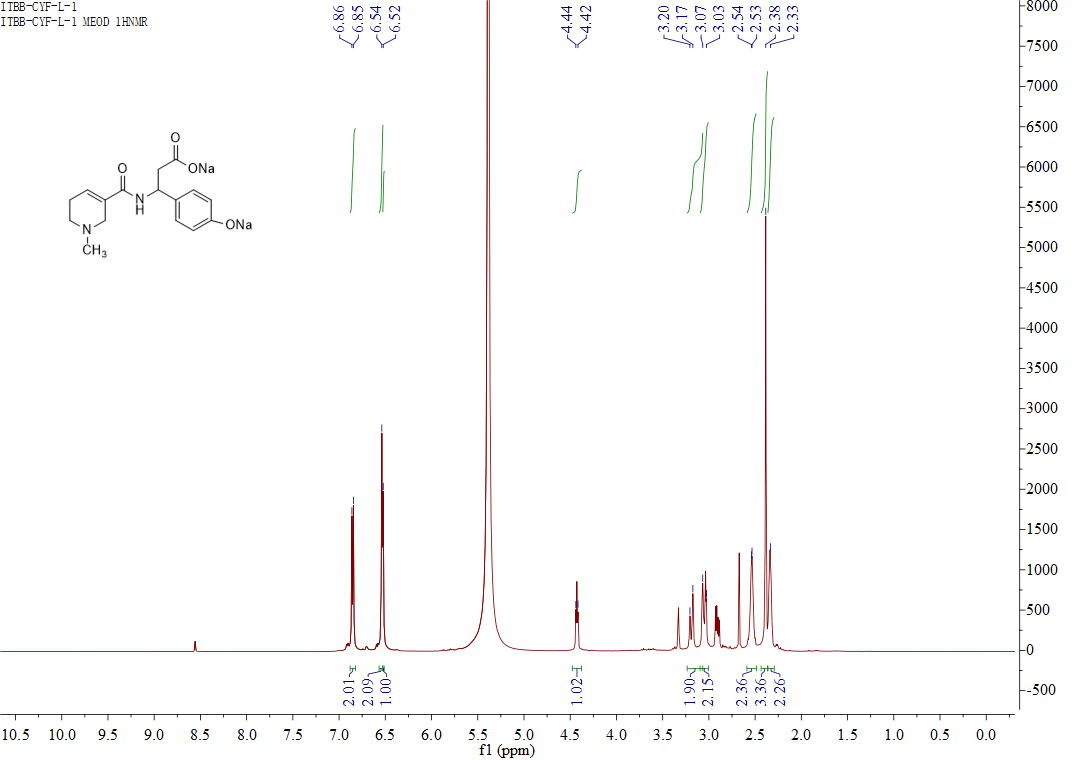


Supplementary Fig.S16 ^1^H NMR spectrum for compound 3h-A


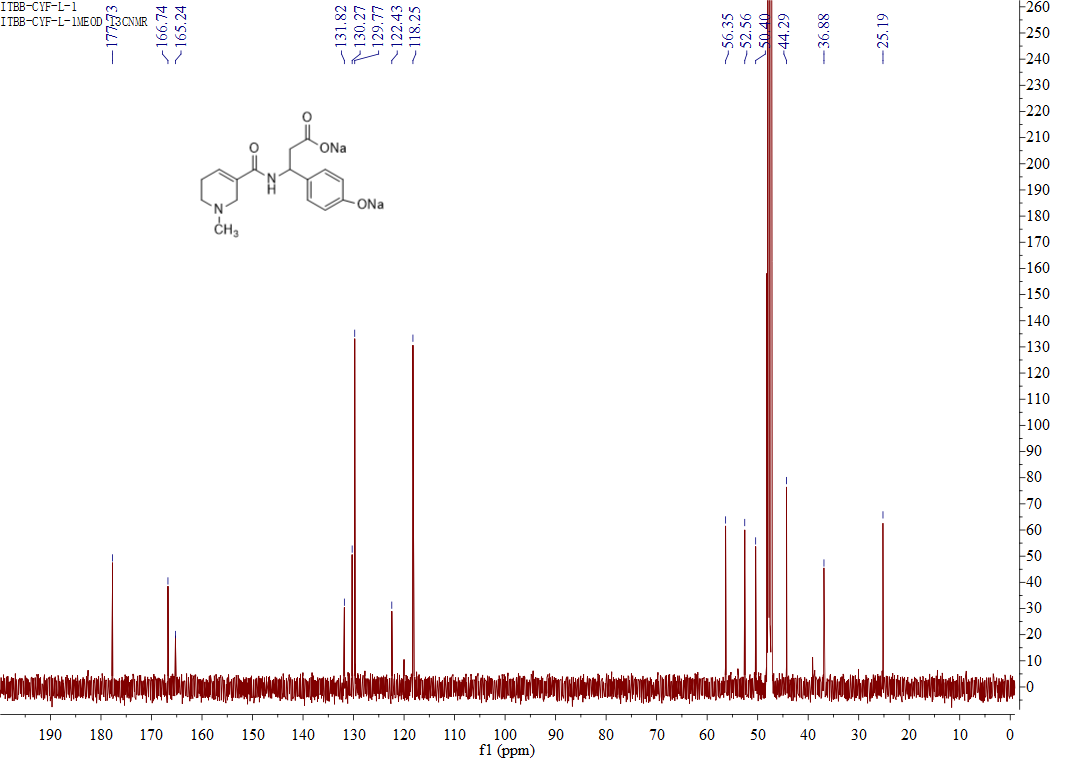


Supplementary Fig.S17 ^13^C-NMR spectrum for compound 3h-B


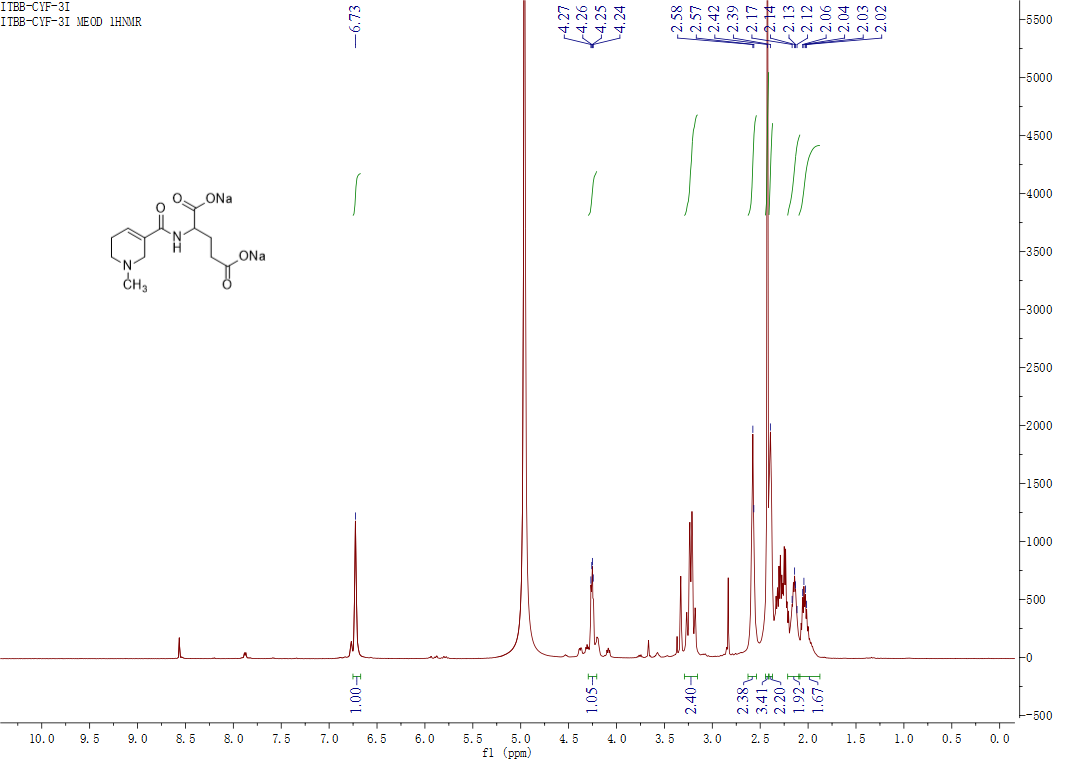
 Supplementary Fig.S18 ^1^H NMR spectrum for compound 3i-A


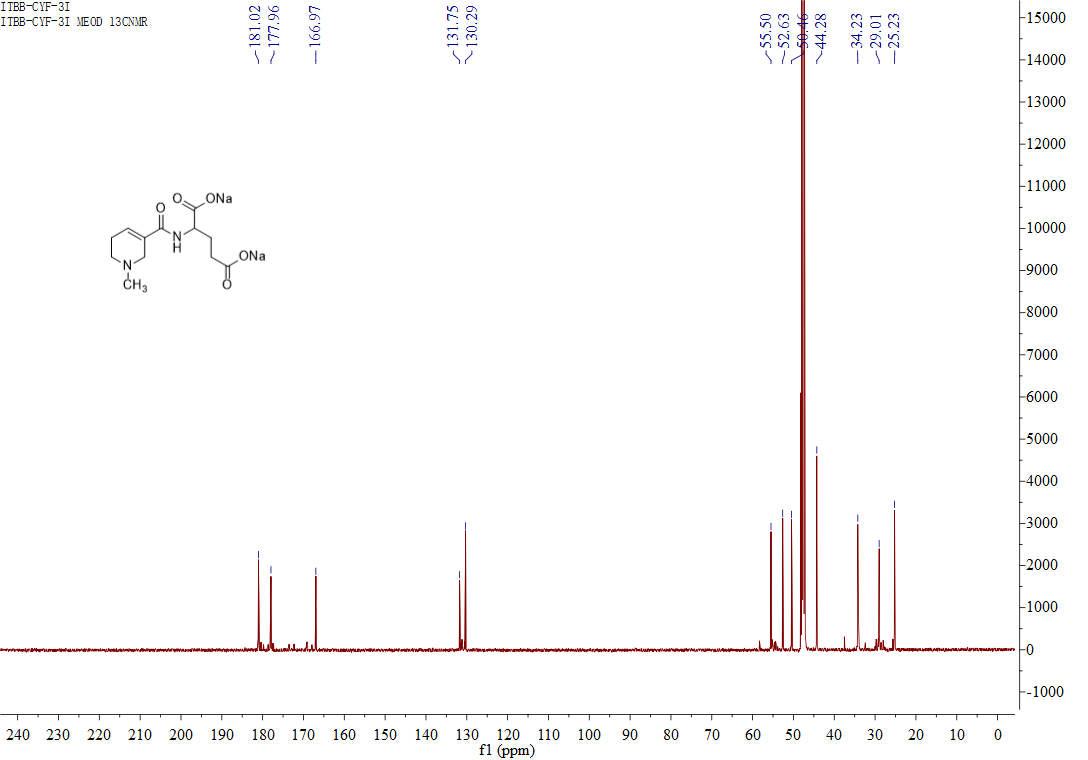


Supplementary Fig.S19 ^13^C-NMR spectrum for compound 3i-B

# 3. HRMS - ESI (m/z) spectrum for target compounds 3a~3i

Supplementary Fig.S20 HRMS - ESI (m/z) spectrum for compounds 3a

Supplementary Fig.S21 HRMS - ESI (m/z) spectrum for compounds 3b

Supplementary Fig.S22 HRMS - ESI (m/z) spectrum for compounds 3c

Supplementary Fig.S23 HRMS - ESI (m/z) spectrum for compounds 3d

Supplementary Fig.S24 HRMS - ESI (m/z) spectrum for compounds 3e

Supplementary Fig.S25 HRMS - ESI (m/z) spectrum for compounds 3f

Supplementary Fig.S26 HRMS - ESI (m/z) spectrum for compounds 3g

Supplementary Fig.S27 HRMS - ESI (m/z) spectrum for compounds 3h

Supplementary Fig.S28 HRMS - ESI (m/z) spectrum for compounds 3i
